# Complete genome sequence data of *Clostridium isatidis* BK32, a lignocellulose-degrading bacterium isolated from compost

**DOI:** 10.1016/j.dib.2025.111874

**Published:** 2025-07-11

**Authors:** Pichpunleu Born, Ayaka Uke, Akihiko Kosugi

**Affiliations:** a Biological Resources and Post-Harvest Division, Japan International Research Center for Agricultural Sciences (JIRCAS), 1-1 Ohwashi, Tsukuba, Ibaraki 305-8686, Japan; b Graduate School of Science and Technology, University of Tsukuba, 1-1-1 Tennodai, Tsukuba, Ibaraki 305-8572, Japan

**Keywords:** Complete genome, Lignocellulose degradation, Glycosyl hydrolase, *Clostridium isatidis*

## Abstract

This data article presents the complete genome sequence of *Clostridium isatidis* BK32, an anaerobic, thermophilic bacterium isolated from compost in Japan. Strain BK32 exhibits enhanced degradation of rice straw compared to the type strain *C. isatidis* DSM15098. Genome sequencing was performed using PacBio Single Molecule Real-Time (SMRT) technology. De novo assembly revealed a single circular chromosome of 2978,451 bp with a GC content of 29.1%. The genome comprises 2769 protein-coding genes and 113 RNA genes. Annotation highlighted 320 genes involved in carbohydrate metabolism. CAZyme analysis identified genes encoding cellulolytic, hemicellulolytic, and amylolytic enzymes. Strain BK32 degraded over 80% of rice straw, underscoring its potential in lignocellulose saccharification. The complete genome sequence is available in the National Center for Biotechnology Information (NCBI) under accession number CP182825.

Specifications Table

**Table:** 

Subject	Biology
Specific subject area	Microbial genomic
Type of data	Tables, Figures
Data collection	Genomic DNA was extracted from a pure culture of strain BK32 using a NucleoBond HMW DNA kit. Genome sequencing via PacBio platforms and Single Molecule Real-Time (SMRT) technologies. The genome was de novo assembled using Flye 2.9. Genome annotation was performed using the Prokaryotic Genome Annotation Pipeline (PGAP). Functional annotation was carried out using the Rapid Annotation System Technology (RAST) server for subsystem features and the dbCAN3 server.
Data source location	Japan International Research Center for Agricultural Sciences (JIRCAS), Tsukuba, Ibaraki, Japan
Data accessibility	Repository name: National Center for Biotechnology Information (NCBI) Data identification number: GenBank accession numbers CP182825, BioProject accession number PRJNA1227611, and Sequence Read Archive (SRA) accession number SRR33781795 Direct URL to GenBank data: https://www.ncbi.nlm.nih.gov/nuccore/2921984449 Direct URL to BioProject data: https://www.ncbi.nlm.nih.gov/bioproject/PRJNA1227611 Direct URL to SRA data: https://www.ncbi.nlm.nih.gov/sra/?term=SRR33781795
Related research article	None

## 1 Value of the Data

• The complete genomic data of *C. isatidis* BK32 suggests a potential candidate for lignocellulose saccharification.
• *C. isatidis* BK32 is the only strain of this species capable of significantly degrading rice straw. Therefore, this sequence data is essential for assessing the biodegradation mechanisms of this strain.
• The genome data of *C. isatidis* BK32 fosters comparative genomic study between *C. isatidis* strains, offering a better understanding of the biological saccharification of lignocellulosic biomass.

## 2 Background

Lignocellulosic biomass is recognized as an alternative energy source due to its renewability, abundance, and affordability [1]. Lignocellulose is a complex biomass consisting of cellulose, hemicellulose, and lignin, which makes it highly resistant and recalcitrant to enzymatic degradation [1,2]. Efficient bioconversion of this biomass requires an organism with a multi-enzyme complex working synergistically to degrade different components [2]. Thus, it is crucial to search for microorganisms that show lignocellulolytic ability.

*Clostridium isatidis* is a Gram-positive, thermophilic, and anaerobic bacillus that belongs to the *Clostridiaceae* family [3]. *C. isatidis* has shown potential for reducing indigo dye, leading to substantial research on its role in the dyeing process [[3], [4], [5], [6]]. However, limited studies have focused on its application in lignocellulose degradation, particularly in rice straw. Among the two species of *C. isatidis*, only strain BK32 shows the ability to saccharify rice straw. Therefore, it is essential to assess its metabolic characteristics through genome sequencing. The current study aims to describe the genomic features of *C. isatidis* BK32 and its repertoire of CAZymes, contributing important genomic information regarding its lignocellulose saccharification potential.

## 3 Data Description

**Fig. 1:**
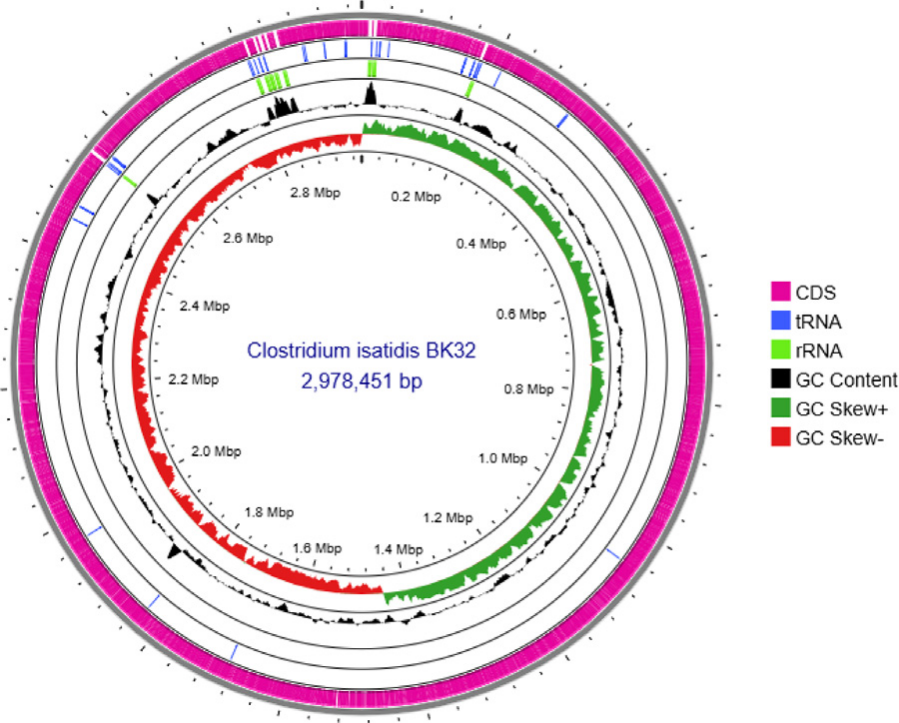
Circular genome map of *Clostridium isatidis* BK32 generated using the CGView server. From the outermost ring inward: backbone (contigs), coding sequences (CDSs), tRNAs, rRNAs, GC content, and GC skew.

This article outlines the complete genomic sequence data of *C. isatidis* BK32, which encodes lignocellulase genes for lignocellulose biomass degradation. The assembled genome resulted in a single contig consisting of 2978,451 base pairs (bp) with a coverage of 354.14X, and the average GC content is 29% (Fig. 1). The genome annotation identified 2769 coding sequences (CDS), 27 rRNA genes, 82 tRNA genes, and 44 pseudo genes (Table 1). The genome of strain *C. isatidis* BK32 has an average nucleotide identity (ANI) value of 99% when compared to *C. isatidis* DSM15098.

**Table 1:** Genome features of *Clostridium isatidis* BK32.

Features	Value
Number of contigs	1
Genome size (bp)	2978,451
N50	2978,451
GC content (%)	29.1
Coverage	354.14
Total genes	2926
Total protein-coding sequences	2813
Functional protein-coding genes	2769
rRNAs	27
tRNAs	82
ncRNAs	4
Total pseudo genes	44

**Fig. 2:**
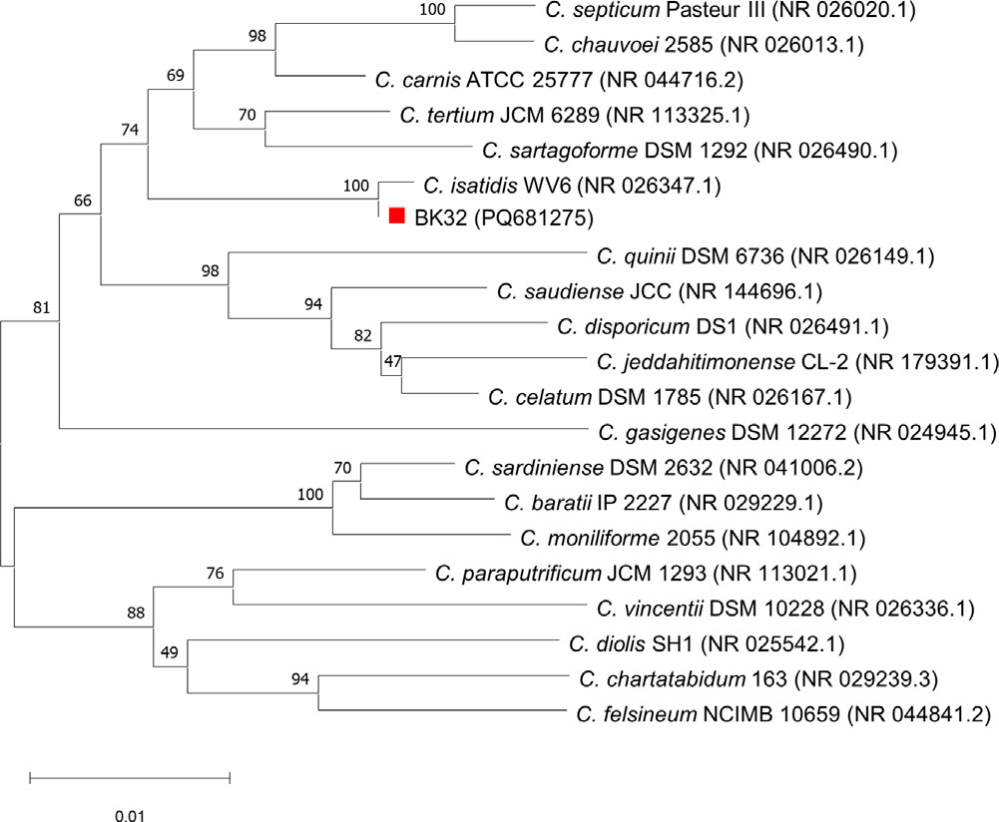
Phylogenetic tree of *Clostridium isatidis* BK32 based on 16S rRNA gene sequences. Bootstrap values (1000 replications) are shown at branch points. The scale bar represents 0.01 substitutions per nucleotide position.

**Fig. 3:**
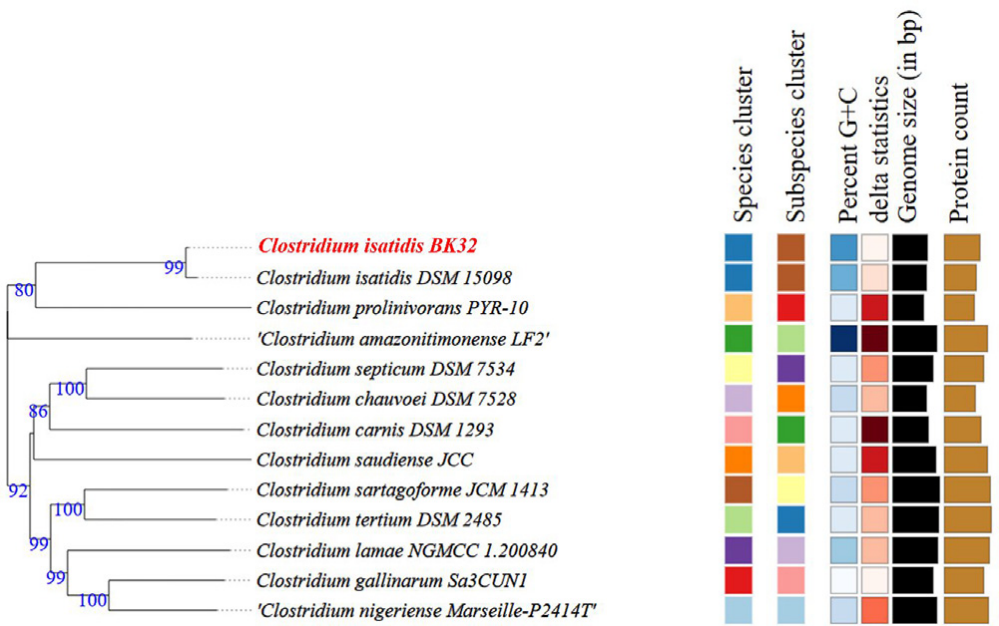
Genome BLAST Distance Phylogeny (GBDP) tree of *Clostridium isatidis* BK32 based on whole-genome sequences, constructed using FastME 2.1.6.1. Branch lengths are scaled according to the GBDP distance formula. Bootstrap support values (>60%) from 100 replications are indicated at branch points, with an average branch support of 90.4%.

The neighbour-joining phylogenetic tree of the 16S rRNA gene indicated that *C. isatidis* is the closest phylogenetic species with 99% sequence similarity (Fig. 2). Furthermore, the phylogenomic analysis via Type (Strain) Genome Server (TYGS) unveiled that strain BK32 is clustered in the same clade as *C. isatidis*, with a digital DNA-DNA hybridization (dDDH) value of 93% (Fig. 3).

**Fig. 4:**
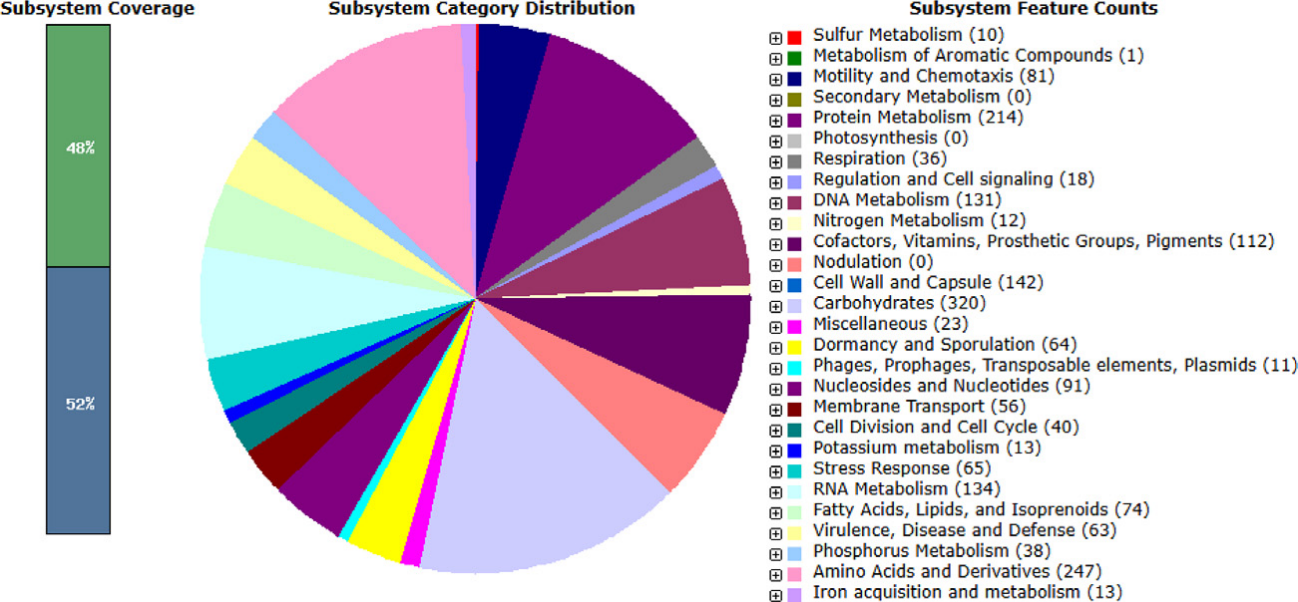
Overview of the subsystem category distribution of protein-coding genes in *Clostridium isatidis* BK32, annotated using the RAST server.

The functional annotation through the Rapid Annotation System Technology (RAST) server for subsystem features predicted 355 subsystems with 48% of subsystem coverage (Fig. 4). The predominant category is carbohydrate metabolism, involving 320 genes, followed by amino acid and derivatives, and protein metabolism with 247 genes and 214 genes, respectively (Fig. 4). Additionally, a total of 85 carbohydrate-active enzymes (CAZymes) were identified from *C. isatidis* BK32, categorised into 5 distinct enzyme classes (Table 2). The strain *C. isatidis* BK32 harbours more genes in the GH family 2, 3, 25, 36, and 112 than the strain *C. isatidis* DSM15098. The predicted enzymes of GH families that catalyse the degradation of carbohydrates include β-(6-phospho)-glucosidase (3.2.1.86), β-glucosidase (3.2.1.21), xylan exo-β−1,4-xylosidase (3.2.1.37), α-galactosidase (3.2.1.22), β-galactosidase (3.2.1.23), amylose endo-α−1,4-glucosidase (3.2.1.1), pullulan endo-α−1,6-glucosidase (3.2.1.41), amylose exo-α−1,4-glucobiosidase (3.2.1.133), and β-N-acetylhexosaminidase (3.2.1.52). The strain BK32 has a higher number of genes associated with the enzymes β-glucosidase (3.2.1.21) and β-N-acetylhexosaminidase (3.2.1.52) compared to strain DSM15098. Moreover, α-galactosidase (3.2.1.22), β-galactosidase (3.2.1.23), and amylose exo-α−1,4-glucobiosidase (3.2.1.133) are absent from the genome of the type strain *C. isatidis* DSM15098. Strain BK32 and DSM15098 were inoculated in BM7 and PYG media, respectively, each supplemented with 1% (w/v) alkali-pretreated rice straw, and incubated anaerobically at 50 °C with shaking at 120 rpm for 8 days to evaluate their biomass saccharification abilities. Following fermentation, residues from both cultures were collected, dried, and their dry weights were measured and subtracted from the initial weights to calculate the extent of rice straw degradation. Strain BK32 achieved >80% substrate degradation, whereas strain DSM15098 was unable to degrade rice straw. This genome annotation data is essential for evaluating the *C. isatidis* BK32 strain's ability to sustainably degrade lignocellulosic biomass.

**Table 2:** Count of carbohydrate-active enzymes (CAZymes) present in the *Clostridium isatidis* BK32 genome compared to DSM15098.

Enzyme class	BK32	DSM15098
Glycoside hydrolases (GHs)	40	36
Glycosyl transferases (GTs)	34	34
Carbohydrate esterases (CEs)	6	7
Carbohydrate-binding modules (CBMs)	2	2
Auxiliary activities (AAs)	3	3
Total number of genes	85	82

In addition to genome sequencing, SMRT sequencing enables the detection of DNA methylation motifs based on sequencing kinetics [7]. The identified methylation motifs were analyzed using the REBASE database to determine their correspondence with known restriction-modification (RM) systems. Among the five detected motifs, four (A, B, C, and D) matched previously characterized recognition sequences associated with Type I, II, and IV RM systems (Table 3). However, no known recognition sequence in REBASE corresponded to motif E (Table 3). Genomic analysis revealed that the putative methyltransferase (MTase) genes responsible for motif E methylation belong to a Type I RM system, comprising three genes encoding a restriction endonuclease (R), a DNA methyltransferase (M), and a specificity subunit (S), designated as CisBK32VP, M.CisBK32V, and S.CisBK32V, respectively. This finding suggests that motif E may represent a novel Type I methylation target.

**Table 3:** Methylation motifs of *C. isatidis* BK32 as assessed by PacBio sequencing.

No.	Motifs (5′−3′)	Modified Position	Methylated Base	Motifs Detected (%)	Number of Motifs Detected	Number of Motifs in Genome	Mean Score	Mean Motif Coverage	Partner Motif
A	GATC	2	m6A	99.95	8096	8100	274.53	99.74	GATC
B	GANTC	2	m6A	99.90	7910	7918	260.33	100.61	GANTC
C	GTATAC	5	m6A	97.39	448	460	208.50	98.84	GTATAC
D1	ACANNNNNNCTC	3	m6A	99.78	454	455	239.54	100.40	GAGNNNNNNTGT
D2	GAGNNNNNNTGT	2	m6A	99.78	454	455	242.47	100.45	ACANNNNNNCTC
E1	CANNNNNNNGTAG	2	m6A	100	530	530	245.72	98.63	CTACNNNNNNNTG
E2	CTACNNNNNNNTG	3	m6A	100	530	530	245.41	98.69	CANNNNNNNGTAG

Motifs A, B, and C are palindromic, while D1 & D2 and E1 & E2 are complementary motifs.

## 4 Experimental Design, Materials and Methods

### 4.1 *Isolation of strain BK32*

*Clostridium isatidis* BK32 was isolated from compost containing rice straw. Briefly, the compost samples were enriched in BM7 medium [8], which contains 2.9 g K_2_HPO_4_, 1.5 g KH_2_PO_4_, 2.1 g urea, 3.0 g yeast extract, 2.5 g Na_2_CO_3_, 0.01 g CaCl_2_·2H_2_O, 2.5 g Na_2_CO_3_, 0.5 g cysteine-HCl, 0.0005 g resazurin, and 200 µl mineral solution (25.0 g MgCl_2_· 6H_2_O, 37.5 g CaCl_2_·2H_2_O, and 0.312 g FeSO_4_·7H_2_O) per liter supplemented with 1% rice straw as the sole carbon source. The medium was boiled and purged with nitrogen gas before autoclaving. The enrichments were incubated at 50 °C for 4 days, and this process was repeated 4 times. Eventually, a pure colony was isolated from the enriched culture using the Hungate roll tube technique [9].

### 4.2 *DNA extraction and genome sequencing*

Strain BK32 was cultured anaerobically in BM7 broth at 50 °C and 120 rpm for 24 h. The extraction of genomic DNA (gDNA) was performed using the NucleoBond HMW DNA kit (Macherey-Nagel, Takara Bio Inc., Kusatsu, Japan) according to the manufacturer’s guidelines. The extracted gDNA was qualified and quantified using a NanoDrop One UV–Vis Spectrophotometer and a Qubit 4.0 Fluorometer (Thermo Fisher Scientific, Waltham, MA, USA). Subsequently, the gDNA was sent to Macrogen Japan Corp. for whole genome sequencing under PacBio RS II system (Pacific Biosciences, Menlo Park, CA, USA). Library preparation was conducted using the SMRTbell Template Prep Kit 1.0 (Pacific Biosciences).

### 4.3 *Genome assembly and annotation*

De novo assembly of the gene sequences were performed using the Flye 2.9 assembly method [10]. The genome completeness and contamination were determined by Benchmarking Universal Single-Copy Orthologs (BUSCO) 5.1.3 [11]. The assembled genome was annotated using the NCBI Prokaryotic Genome Annotation Pipeline (PGAP) [12] and RAST for gene mapping to subsystems [13]. The dbCAN3 server was used to analyse the carbohydrate-active enzymes (CAZymes) [14]. The genome's circular map was generated using CGView [15]. Putative restriction-modification (RM) systems were identified through the Restriction Enzyme database (REBASE) [16].

### 4.4 *Phylogenomic classification*

The 16S rRNA sequences from closely related taxa were obtained through BLAST searches against the GenBank database and aligned with the 16S rRNA sequence of strain BK32 using CLUSTAL_W in MEGA 11 [17]. The neighbour-joining phylogenetic tree was constructed with 1000 bootstrap replications in MEGA 11. Taxonomic analysis based on whole genomes was analysed using Genome BLAST Distance Phylogeny (GBDP) in the Type (Strain) Genome Server (TYGS) [18].

### 4.5 *Rice straw saccharification*

The rice straw used in this study was purchased from Miyahara store (Nagano, Japan). The biomass was milled to a fine powder using a 0.5 mm mesh screen (ZM-100; Retsch, Haan, Germany), and subsequently subjected to alkaline pretreatment as described by Tachaapaikoon et al. [19]. For the biodegradation assay, subcultures of strains BK32 and DSM15098, each prepared from 3-day-old cultures (1% v/v inoculum), were inoculated into 15 mL of BM7 or PYG medium, respectively, containing 1% (w/v) alkali-pretreated rice straw and purged with N₂ gas. Cultures were incubated anaerobically at 50 °C with shaking at 120 rpm for 8 days. After incubation, the residual biomass was collected by centrifugation at 10,000 × *g* for 10 min, dried at 70 °C for 2 days, and weighed. The extent of rice straw degradation was determined by comparing the dry weight of the residual biomass to that of an uninoculated control. The percentage of substrate degradation was calculated as the reduction in dry weight relative to the initial amount, as previously described by Shikata et al. [20].

## Limitations

Not applicable.

## Ethics Statement

The authors have read and follow the ethical requirements for publication in Data in Brief and confirming that the current work does not involve human subjects, animal experiments, or any data collected from social media platforms.

## CRediT Author Statement

**Pichpunleu BORN:** Conceptualization, Methodology, Data curation, Draft writing. **Ayaka UKE:** Methodology, Investigation, Writing- Reviewing and Editing. **Akihiko KOSUGI:** Supervision, Writing- Reviewing and Editing.

## Data Availability

NCBIBioProject accession number PRJNA1227611 (Original data). NCBIGenBank accession numbers CP182825 (Original data).
